# Exploring the Association Between Clinical Features and CBCT Findings in TMJ Degenerative Joint Disease

**DOI:** 10.1111/joor.13970

**Published:** 2025-04-15

**Authors:** Michael Wu, Hollis Lai, Fabiana T. Almeida, Reid Friesen

**Affiliations:** ^1^ Mike Petryk School of Dentistry Faculty of Medicine and Dentistry, University of Alberta Edmonton Alberta Canada

**Keywords:** clinical signs, cone‐beam computed tomography, degenerative joint disease, imaging, temporomandibular disorders, TMJ

## Abstract

**Background:**

Temporomandibular joint (TMJ) degenerative joint disease (DJD) involves progressive osseous changes and is commonly associated with temporomandibular disorders (TMD). Cone‐beam computed tomography (CBCT) is a valuable diagnostic tool for evaluating these changes. However, the relationship between clinical signs and symptoms, such as TMJ clicking or pain and radiographic findings remains poorly understood. Clarifying these associations can refine imaging prescribing practices and improve patient‐specific diagnostic strategies.

**Objective:**

This study aimed to investigate the association between clinical signs and symptoms of TMD and radiographic features of TMJ DJD detected on CBCT, emphasising its diagnostic value and limitations.

**Methods:**

A retrospective chart review of 98 patients (196 TMJs) was conducted at a university‐based oral medicine clinic. Clinical signs, including TMJ clicking, muscle pain and joint pain, were documented and CBCT findings, such as osteophytes and erosions, were analysed. Logistic regression was used to assess associations.

**Results:**

A significant association was identified between TMJ clicking and the presence of osteophytes (*p* < 0.05). No significant associations were observed between other clinical features, including muscle and joint pain and CBCT findings.

**Conclusion:**

The findings support an indication‐driven approach to CBCT imaging, highlighting its diagnostic value in patients with specific clinical presentations, such as TMJ clicking, combined with additional clinical indicators. Routine CBCT imaging for all patients with TMD is not justified and future research should focus on refining imaging guidelines to ensure judicious use in TMJ diagnostics.

## Graphical abstract

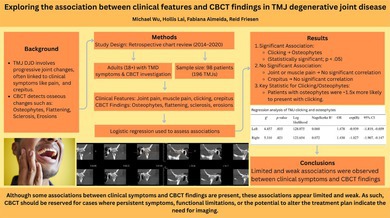

## 1 Introduction

According to the diagnostic criteria for temporomandibular disorders (TMD) for clinical and research applications (DC/TMD), pathologic changes in the temporomandibular joint (TMJ) may indicate the presence of degenerative joint disease (DJD) [1]. DJD, or osteoarthritis, occurs when the articular cartilage in the TMJ deteriorates, and osseous changes in the mandibular condyle or the temporal bone structures occur [2, 3]. While DJD is sometimes described as a progressive breakdown of the TMJ, it is important to recognise that not all osseous changes are signs of active disease. The TMJ can adapt and remodel in response to changes in loading, and this process can sometimes resemble degenerative changes. For example, features like condylar flattening or changes to the articular eminence shape may reflect remodelling rather than true DJD. This distinction is important when interpreting CBCT findings. Remodelling changes are often painless, while true DJD may or may not cause symptoms [2, 4].

Based on the patient's history and clinical presentation, the presence of joint noise—either as described in the patient's history in accordance with the DC/TMD criteria or as a clinical finding of crepitus detected during functional movement examination—is considered diagnostic for DJD [1]. According to the DC/TMD guidelines, crepitus is defined as a coarse, grating, or crunching sound that may be detected through palpation during mandibular movement, typically indicative of bony changes in the TMJ. This differs from clicking, which is characterised by a distinct, sharp sound often associated with disc displacement. Without imaging, the diagnostic validity is relatively low, as imaging serves as the reference standard for confirming the diagnosis. Radiographic imaging is particularly crucial for verifying the diagnosis of DJD when indicated [1, 4]. Cone‐beam computed tomography (CBCT) is the reference standard for assessing osseous changes, given proper selection criteria. The acronym ALADAIP (as low as diagnostically acceptable, indication‐oriented and patient‐specific) should be the target when treatment planning and imaging [5].

**FIGURE 1:**
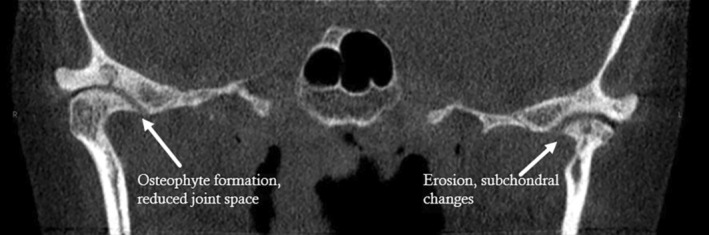
Coronal CBCT view of TMJ with degenerative changes. Coronal view of the TMJ displaying osteophyte formation, reduced joint space and subchondral changes in the articular eminence and right condyle. The left TMJ exhibits cortical erosion and subchondral changes.

**FIGURE 2:**
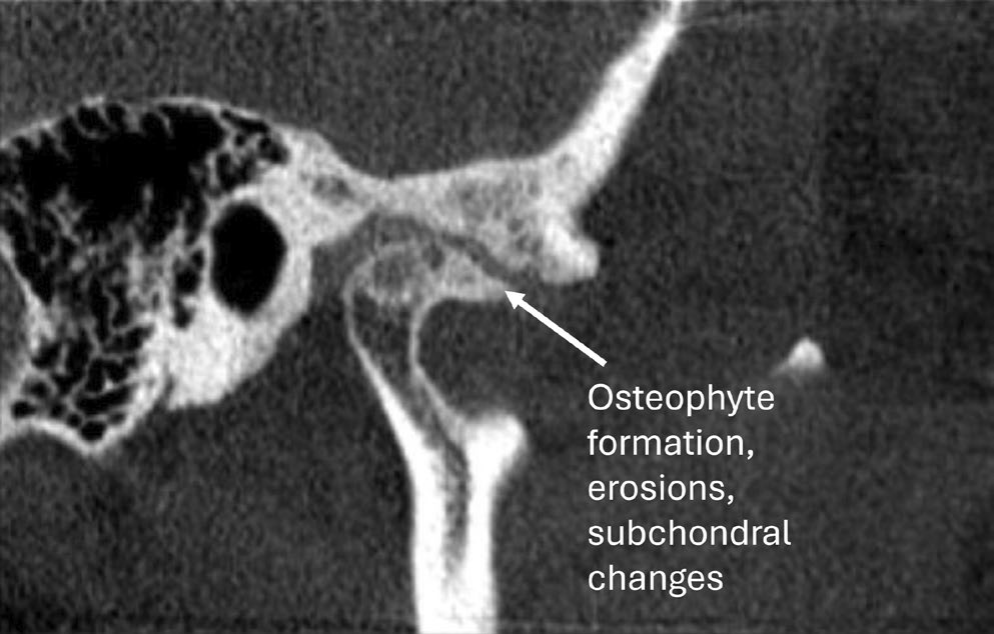
Sagittal CBCT view of right TMJ with osteophyte formation. Sagittal view showing osteophyte formation in the right condyle, along with erosion and subchondral changes in both the condyle and articular eminence.

**FIGURE 3:**
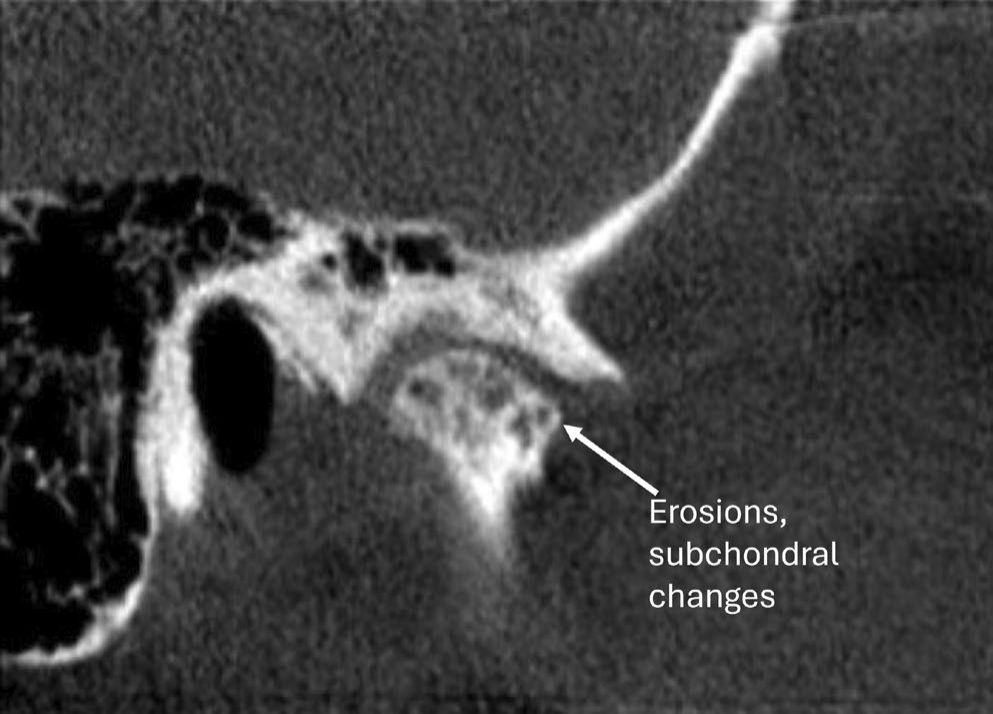
Sagittal CBCT view of left TMJ with degenerative changes. Sagittal view showing condylar erosion and subchondral changes in the articular eminence of the left TMJ.

Various factors such as age, gender, trauma, parafunctional habits, systemic disease and disc displacement can increase the incidence of osseous abnormalities. However, joint overloading is considered the most common local cause [6, 7]. Aside from joint noises, common signs and symptoms of TMDs include TMJ pain, masticatory muscle pain and limited range of motion [8]. Osseous changes of DJD noted radiographically include subchondral cysts, erosions of the articular surfaces, generalised subchondral sclerosis and osteophytes; at least one must be present when a diagnosis of DJD needs to be confirmed [1] (Figures 1, 2, 3).

Temporomandibular joint degenerative joint disease is generally broken down into three stages: early stage (involving noises and dysfunction), intermediate stage (characterised by TMJ destruction and often associated with pain) and late phase (associated with joint ‘burnout’ and stabilisation of the joints) [9].

Temporomandibular disorders have an estimated prevalence of 31% in the adult population, with DJD being the most common joint pathology affecting the TMJs [2, 10]. Thus, understanding clinical factors that predict radiographic changes can help to identify TMDs and DJD earlier and aid optimal management of these disease processes. While CBCT is a well‐established and reliable tool for identifying intra‐articular osseous changes in TMJ disorders, its role in correlating radiographic findings with specific clinical signs and symptoms remains less explored. This study focuses on identifying such associations to refine the clinical utility of CBCT in targeted diagnostic and therapeutic contexts. Given the diagnostic challenges in correlating clinical findings with radiographic evidence in TMJ disorders, this study aims to explore the associations between specific clinical signs and symptoms and radiographic findings indicative of DJD. The study seeks to clarify the utility of CBCT imaging in identifying radiographic changes that correspond with clinical presentations, thereby contributing to more indication‐driven and patient‐specific diagnostic criteria.

## 2 Materials and Methods

This retrospective chart review study was conducted following the University of Alberta Health Research Ethics Panel approval (Pro00095294). The study followed STROBE guidelines for reporting observational studies.

To collect data, the chart records and CBCT reports were reviewed on all patients seen by the Oral Medicine clinic, Mike Petryk School of Dentistry, University of Alberta over 7 years, from January 1, 2014 to December 31, 2020. All patients included in this study have consented to their involvement in research at the school as part of their intake at the clinic.

Inclusion criteria:

- Patients referred to and seen at the University of Alberta Oral Medicine clinic for TMD symptoms.
- 18 and over.
- Patients who had a CBCT of TMJ taken as part of their TMD assessment.

Exclusion criteria:

- Patients under the age of 18.
- Patients in which CBCT demonstrates compromised image quality interfering with the interpretation.
- History of jaw surgery/condylar fracture.
- History of connective tissue disease/rheumatologic conditions including but not limited to rheumatoid arthritis; idiopathic condylar resorption; TMJ condylar hyperplasia; and orofacial syndromic patients.

Existing clinical data collected from the chart records were included, with no patients contacted for follow‐up questioning. Each chart included clinical findings documented by oral medicine residents and corroborated by dental specialists. DC/TMD criteria are utilised in the clinic with calibrated residents utilising the DC/TMD examination template to standardise data collection [1]. Clinical data were collected, ensuring standardised assessment across all evaluated features. This included documenting pain on palpation of the masseter, temporalis and lateral TMJ capsule, as well as recording joint noises such as clicking and crepitus as separate variables. Clicking was defined as a distinct, audible noise occurring during jaw movement, while crepitus was defined as a continuous, grating, grinding, or crackling sound with jaw movement. Both patient‐reported symptoms and clinician‐confirmed findings were documented and clicking and crepitus were analysed independently.

All patients in this study underwent their CBCT examination at the Mike Petryk School of Dentistry—University of Alberta, under a consistent protocol. Patients were scanned using an ICAT CBCT unit (Imaging Sciences International, Hatfield, PA, USA), with parameters: medium FOV TMJ scan, 0.2 mm voxel size, 120 Kvp and 5 mA. Radiographic data were collected from the CBCT reports completed by two calibrated oral and maxillofacial radiologists. Axial, sagittal, coronal views and TMJ multiplanar reconstruction were used. Bias was reduced by blinding all data acquisition when possible. The clinicians screened patients as to the requirement and utility of CBCT imaging to help guide diagnosis and subsequent treatment. Not all patients seen in the clinic received routine CBCT imaging and a panoramic radiograph was more routinely taken as an initial screening tool. Any and all patients meeting exclusion criteria were excluded from data collection both clinically and radiographically.

### 2.1 Statistical Methods

A quantitative analysis of the association (linear or non‐linear relationship) between specific clinical symptoms and CBCT findings was conducted. The following categories were coded to extract the data from the clinical findings: age, biological sex, joint pain, masticatory muscle pain, clicking and crepitus. In addition, the following radiographic findings were coded: mandibular condyle (MC) and articular eminence (AE) flattening, presence of osteophytes, joint space narrowing, cortical erosions, subchondral sclerosis and subcortical erosions and cysts. The entirety of the mandibular condyle was assessed, including the superior surface as well as the anterior, posterior, medial and lateral poles.

Descriptive measures (mean ± SD, medians and ranges) were calculated for each variable. Clinical and radiographic features were analysed and compared using Statistical Package for Social Sciences—SPSS (Version 28, IBM, Armonk, NY, USA). Logistic regression evaluated the association between clinical signs/symptoms and radiographic features.

## 3 Results

Ninety‐eight patient charts (196 TMJs) with adequate clinical and radiographic records were reviewed. All findings were differentiated into left and right to facilitate statistical analysis associating clinical signs/symptoms with radiographic findings. There was no missing data noted.

### 3.1 Study Characteristics

Across the 98 patients, 20 males and 78 females were included, with an age range of 18–83 years and a median age of 50.5 years.

### 3.2 Clinical Features

The assessed clinical features included arthralgia, masticatory myalgia, clicking and crepitus. The majority of patients presented with arthralgia (95%), myalgia (68%) and TMJ clicking (70%), while only 32% of patients presented with crepitus (Table 1).

**TABLE 1:** Clinical findings and frequency of positive and negative reports.

Clinical findings	Positive	Negative	Percent
Right joint pain (reported and clinical)	79	19	81%
Left joint pain (reported and clinical)	82	16	84%
Right muscle pain (masseter + temporalis)	57	41	58%
Left muscle pain (masseter + temporalis)	61	37	62%
Right clicks (reported + clinical)	60	38	61%
Left clicks (reported + clinical)	58	40	59%
Right crepitus (reported + clinical)	30	68	31%
Left crepitus (reported + clinical)	31	67	32%

### 3.3 Radiographic Features

The majority of patients were found to have MC + AE flattening (71%) and MC/AE subchondral sclerosis (61%) (Table 2).

**TABLE 2:** Radiographic findings and frequency of positive and negative observations.

Radiographic findings	Positive	Negative	Percent
Right MC + AE flattening	62	36	63%
Left MC + AE flattening	63	35	64%
Right osteophytes	31	67	32%
Left osteophytes	30	68	31%
Right narrow joint space	28	70	29%
Left narrow joint space	28	70	29%
Right cortical erosion (MC/AE)	31	67	32%
Left cortical erosion (MC/AE)	35	63	36%
Right MC/AE subchondral sclerosis	50	48	51%
Left MC/AE subchondral sclerosis	49	49	50%
Right subcortical erosions (erosions/cysts in MC/AE)	22	76	22%
Left subcortical erosions (erosions/cysts in MC/AE)	24	74	24%

### 3.4 Logistic Regression

The Phi coefficient was calculated for correlation, but no significant correlations were noted between joint or muscle pain and radiographic features on either side and crepitus and radiographic features on either side. Eight binary logistic regressions were conducted on the four clinical findings. Logistic regression models were created using six radiographic features to predict clinical findings on each side of the TMJ. Of the eight models tested, only two showed significant results and are presented. Both clicking and crepitus were analysed as independent variables; clicking demonstrated a significant association with osteophyte formation, while crepitus showed no significant associations with radiographic features.

The logistic regression model for clicks on the left side was statistically significant (*X* ^2^ = 4.457, df = 1, *p* = 0.035), where the model with log‐likelihood of 128.075 had a Nagelkerke *R* ^2^ of 0.06 and accurately predicted 63.3% of cases presenting with left osteophytes for left clicks. This means that those patients presenting with osteophytes were 1.478 times as likely to present with clicks on the left side (exp(*B*) = −0.939, 95% CI [−1.819, −0.059]). Similarly, the logistic regression model for clicks on the right side was also statistically significant (*X* ^2^ = 5.310, df = 1, *p* = 0.021), where the model with log‐likelihood of 123.654 accounted for 7.2% of the variance, as demonstrated by the Nagelkerke *R* ^2^ and accurately predicted 64.9% of cases presenting with right osteophytes for right clicks. Those patients presenting with osteophytes were 1.430 times as likely to present with clicks on the right side (exp(*B*) = −1.027, 95% CI [−1.907, −0.147]). All other radiographic findings were not associated with clicking of the TMJ. The results of the statistically significant regressions are presented in Table 3. Although crepitus is considered the defining clinical finding for TMJ DJD, no statistically significant associations were found after conducting the logistic regression, hence its exclusion from Table 3.

**TABLE 3:** Regression analysis of TMJ clicking and osteophytes.

	*χ* ^2^	*p*	Log likelihood	Nagelkerke *R* ^2^	OR	Exp(B)	95% CI
Left	4.457	0.035	128.075	0.060	1.478	−0.939	−1.819, −0.059
Right	5.310	0.021	123.654	0.072	1.430	−1.027	−1.907, −0.147

## 4 Discussion

This study examined the association between clinical signs and symptoms of TMJ disorders and radiographic findings on CBCT, with a focus on DJD. A significant association was identified between TMJ clicking and the presence of osteophytes, with 31% of left joints and 32% of right joints showing osteophyte formation. However, no significant associations were observed between other clinical features, such as joint pain, muscle pain, or crepitus and radiographic findings, as confirmed by the low prevalence of cortical erosions, subcortical cysts and narrowed joint space in both symptomatic and asymptomatic joints. These results align with previous research reporting weak or no association between clinical symptoms and radiographic changes [11]. Although crepitus is recognised as a diagnostic feature of DJD in the DC/TMD criteria, our analysis did not identify any significant associations between crepitus and radiographic findings. This may be due to sample variability, study limitations, or the possibility that crepitus reflects a different stage or pattern of DJD progression than we assessed. Regardless, this finding highlights the complexity of diagnosing DJD, where clinical signs may not always align with imaging results.

Our study identified a significant association between TMJ clicking and osteophyte formation, a recognised radiographic feature of DJD in the DC/TMD criteria. This suggests that clicking may indicate structural changes within the TMJ's osseous structures. While both clicking and crepitus were analysed independently, only clicking showed a significant association with osteophytes, underscoring the need for further research to better understand the distinct clinical implications of these joint noises and their roles in DJD progression.

Although osteophytes are a recognised DJD feature, their link to clicking in our study suggests a possible overlap with internal derangement mechanisms. While clicking is traditionally associated with disc displacement with reduction, its connection to osteophytes raises questions about whether osteophyte formation reflects a shared or overlapping process and if osseous changes follow disc displacement, or possibly vice versa. Our study did not assess the size or location of osteophytes, which may influence their clinical relevance. Additionally, without MRI, we could not evaluate soft tissue changes or disc position.

Given that TMJ clicking occurs in up to 20% of the population and is not always indicative of pathology, it cannot independently justify CBCT imaging [12]. Instead, this association may be suggestive of overlap with concomitant and benign disc displacement. These results emphasise the need for an indication‐driven approach, reserving CBCT for cases where imaging informs treatment planning or surgical decision‐making. While CBCT effectively visualises osseous changes, it cannot evaluate soft tissue structures such as the articular disc, which may play a key role in the early stages of DJD. MRI remains the reference standard for soft tissue evaluation and should complement CBCT in selected cases requiring detailed assessment of joint function and anatomy [13]. Although MRI is more costly and less accessible, its superior ability to assess soft tissue structures highlights its importance in specific cases where detailed diagnostic information is needed.

Interestingly, the lack of association between radiographic findings and pain symptoms aligns with prior studies. For instance, cortical erosion was present in 34% of joints, yet it did not correlate with reports of joint or muscle pain. Similarly, subchondral sclerosis, observed in approximately half of the joints, did not align with reported symptoms. These findings highlight the complexity of TMJ disorders, where pain may arise from factors beyond structural changes, including functional and psychosocial influences, as found in other papers [14]. The variability in symptom presentation underscores the need for a multimodal approach to TMJ evaluation, integrating clinical examination, imaging and patient‐reported outcomes.

The results also reveal the diagnostic challenges of DJD at different stages. Early‐stage DJD changes are not easily detectable on CBCT. While osteophytes, subchondral sclerosis and other advanced changes were documented, the study did not differentiate between active and stable phases of DJD. These findings reinforce the need for complementary imaging tools and targeted research to better understand disease progression and its clinical implications. Dynamic imaging modalities, such as bone scans, have been proposed to provide additional insights into active inflammation but are associated with higher radiation exposure and limited accessibility [15].

The retrospective design of this study introduces inherent limitations, including variability in chart documentation and potential bias in CBCT interpretation, despite efforts to calibrate radiologist assessments. The exclusion of asymptomatic individuals, though not explicitly stated in the exclusion criteria, is implied by the fact that all patients were evaluated in the Orofacial Pain/TMD clinic, suggesting the presence of some form of concern. This limitation affects the generalisability of the findings, as degenerative changes are commonly observed in individuals without symptoms [15] Additionally, the cross‐sectional nature of the study precludes establishing causation or evaluating temporal relationships between clinical symptoms and radiographic findings.

This study underscores that CBCT should not be routinely employed or used for all patients presenting with TMJ pain. Instead, CBCT use must be indication‐driven, focusing on minimising radiation exposure in line with ALADAIP principles. Future research with the aid of MRI to evaluate soft tissue changes may provide further insight into the possible relationship between internal derangement and degenerative changes—osteophytes in particular. Studies comparing symptoms of DJD with CBCT and MRI findings together may also reveal relationships between osteophyte characteristics, such as size and location and clinical findings. Additionally, broader research, including both symptomatic and asymptomatic individuals, would help clarify the full spectrum of TMJ disorders and improve generalisability. Prospective longitudinal studies using standardised clinical and radiographic measures to track DJD progression and the predictive value of early clinical signs are essential for enhancing the reliability of findings and gaining a better understanding of disease progression. Additionally, the inclusion of a well‐defined control group, such as asymptomatic individuals or those without imaging evidence of TMJ pathology, would enhance the validity and applicability of future findings. Standardised tools to measure pain would be recommended to strengthen future studies [16, 17]. Other aspects of pain, such as psychosocial factors and inflammatory mediators, should also be considered to provide a holistic understanding of TMJ disorders. Furthermore, standardised recording methods for clinical and radiographic measures would minimise heterogeneity in reporting and improve data reliability.

## 5 Conclusion

This study demonstrates a significant association between TMJ clicking and osteophytes, while limited or no associations were observed between other clinical features, such as joint or muscle pain and CBCT findings. These results highlight that CBCT imaging should not be used routinely for patients presenting with TMD pain alone. Instead, CBCT should be reserved for cases where clinical findings, such as persistent symptoms or functional limitations, indicate the potential for osseous abnormalities that may influence diagnosis or treatment planning. Complementing CBCT with MRI for definitive soft tissue imaging is important, particularly in cases where evaluation of the articular disc or joint capsule is necessary. Together, these imaging modalities can provide a more comprehensive assessment of TMJ disorders.

## Author Contributions

**Michael Wu:** conceptualisation, methodology, formal analysis, investigation, data curation, writing – original draft. **Hollis Lai:** methodology, formal analysis, writing – review and editing. **Fabiana T. Almeida:** conceptualisation, methodology, formal analysis, investigation, data curation, writing – original draft, writing – review and editing, supervision, project administration, funding acquisition. **Reid Friesen:** conceptualisation, methodology, formal analysis, investigation, data curation, writing – original draft, writing – review and editing, supervision, project administration, funding acquisition.

## Conflicts of Interest

The authors declare no conflicts of interest.

## Peer Review

The peer review history for this article is available at https://www.webofscience.com/api/gateway/wos/peer‐review/10.1111/joor.13970.

## Supporting information


Data S1.

## Data Availability

Data analysis available on request from the authors.
