# Supplementary figures and images for: Exploring the Association Between Clinical Features and CBCT Findings in TMJ Degenerative Joint Disease
Source: J Oral Rehabil. 2025 Apr 15;52(7):1043–9. doi: 10.1111/joor.13970 (PMC12162413; doi:10.1111/joor.13970)

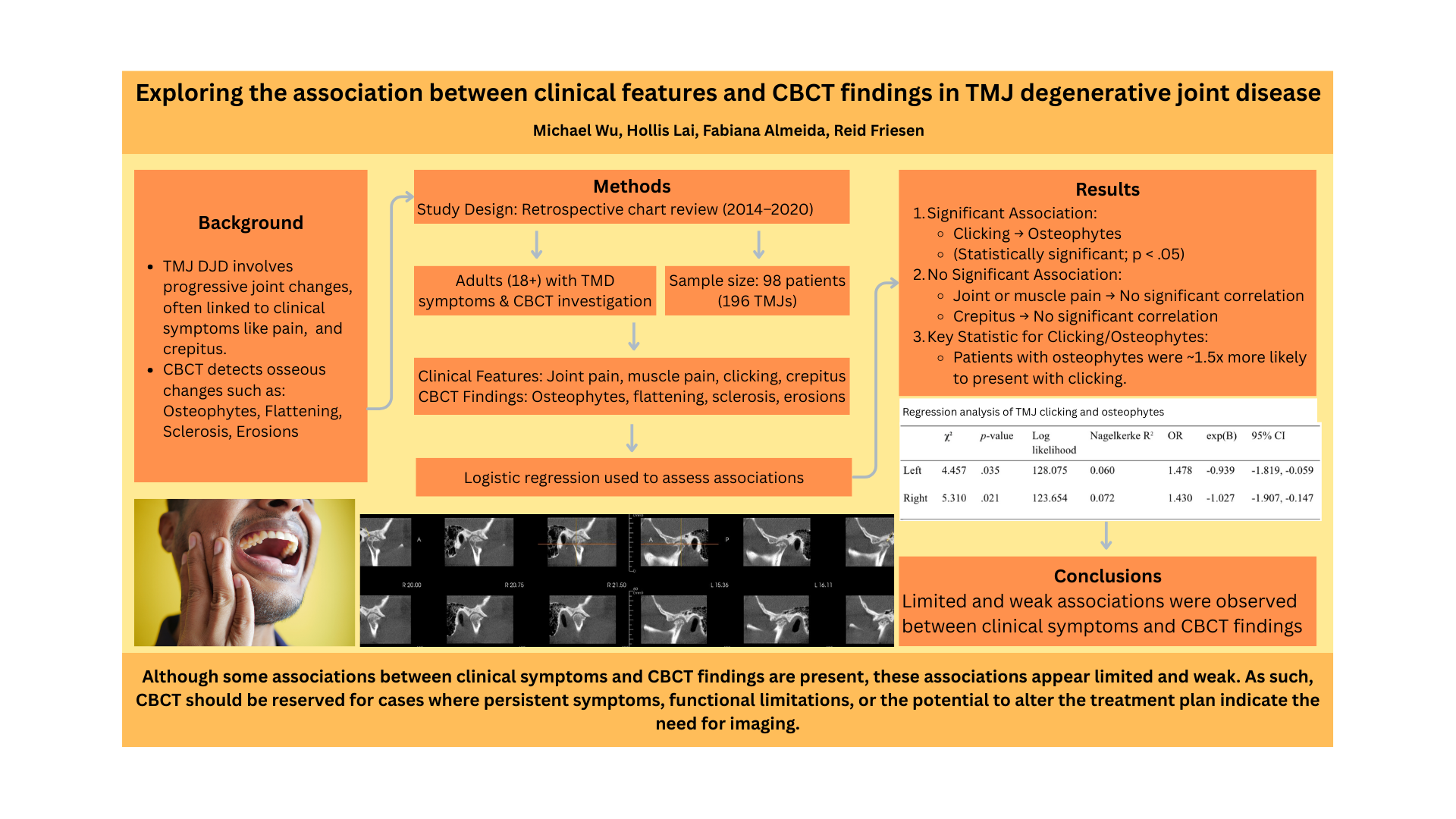

Supplement: Supplementary file 1 — Data S1. [file JOOR-52-1043-s001.tiff]
